# Supplementary material for: ‘Missingness’ in health care: Associations between hospital utilization and missed appointments in general practice. A retrospective cohort study
Source: PLoS One. 2021 Jun 24;16(6):e0253163. doi: 10.1371/journal.pone.0253163 (PMC8224850; doi:10.1371/journal.pone.0253163)
Supplement: S1 File — (DOCX) [file pone.0253163.s001.docx]

| 04/03/2020 17:44 | |
| --- | --- |
|  | |
| SMR 00 hospital specialty category | |
|  | |
| **Name** | **Coded Text** |
| Adult Medicine | A1 General Medicine |
| Adult Medicine | A11 Acute Medicine |
| Adult Medicine | A2 Cardiology |
| Adult Medicine | A3 Clinical Genetics |
| Adult Medicine | A4 Tropical Medicine |
| Adult Medicine | A5 Clinical Pharmacology & Therapeutics |
| Adult Medicine | A6 Infectious Diseases |
| Adult Medicine | A7 Dermatology |
| Adult Medicine | A8 Endocrinology & Diabetes |
| Adult Medicine | A81 Endocrinology |
| Adult Medicine | A82 Diabetes |
| Adult Medicine | A9 Gastroenterology |
| Adult Medicine | AA Genito-Urinary Medicine |
| Adult Medicine | AB Geriatric Medicine |
| Adult Medicine | AC Homeopathy |
| Adult Medicine | AD Medical Oncology |
| Adult Medicine | AG Renal Medicine |
| Adult Medicine | AH Neurology |
| Adult Medicine | AJ Integrative Care |
| Adult Medicine | AK Occupational Medicine |
| Adult Medicine | AM Palliative Medicine |
| Adult Medicine | AN Public Health Medicine |
| Adult Medicine | AP Rehabilitation Medicine |
| Adult Medicine | AQ Respiratory Medicine |
| Adult Medicine | AR Rheumatology |
| Adult Medicine | AS Sport & Exercise Medicine |
| Adult Medicine | AT Medical Ophthalmology |
| Adult Medicine | AV Clinical Neurophysiology |
| Adult Medicine | AW Allergy |
| Adult Medicine | C31 Pain Management |
|  | |
| Reports\\SMA hospital specialty caetgory Final | Page 1 of 6 |
| 04/03/2020 17:44 | |
|  | |
|  | |
| **Name** | **Coded Text** |
| Adult Medicine | C51 Audiological Medicine |
| Adult Medicine | CC Intensive Care Medicine |
| Adult Medicine | D1 Community Dental Practice |
| Adult Medicine | D2 General Dental Practice |
| Adult Medicine | D4 Oral Medicine |
| Adult Medicine | D5 Orthodontics |
| Adult Medicine | D7 Dental Public Health |
| Adult Medicine | D9 Oral Pathology |
| Adult Medicine | DA Oral Microbiology |
| Adult Medicine | DB Dental & Maxillofacial Radiology |
| Adult Medicine | DE Special Care Dentistry |
| Adult Medicine | E1 General Practice |
| Adult Medicine | H1 Clinical Radiology |
| Adult Medicine | H1A Breast Screening Service |
| Adult Medicine | H2 Clinical Oncology |
| Adult Medicine | J1 Histopathology |
| Adult Medicine | J2 Blood Transfusion |
| Adult Medicine | J3 Chemical Pathology |
| Adult Medicine | J4 Haematology |
| Adult Medicine | J5 Immunology |
| Adult Medicine | J6 Medical Microbiology & Virology |
| Adult Medicine | J61 Microbiology |
| Adult Medicine | J62 Virology |
| Adult Medicine | J7 Diagnostic Neuropathology |
| Adult Medicine | J8 Forensic Histopathology |
| Adult Medicine | R1 Chiropody/Podiatry |
| Adult Medicine | R3 Dietetics |
| Adult Medicine | R4 Occupational Therapy |
| Adult Medicine | R41 Industrial therapists |
| Adult Medicine | R5 Physiotherapy |
| Adult Medicine | R6 Speech and Language Therapy |
|  | |
| Reports\\SMA hospital specialty caetgory Final | Page 2 of 6 |
| 04/03/2020 17:44 | |
|  | |
|  | |
| **Name** | **Coded Text** |
| Adult Medicine | R8 Audiological science |
| Adult Medicine | R81 Hearing aids |
| Adult Medicine | R82 Audiometry |
| Adult Medicine | R9 Medical physics |
| Adult Medicine | RA Pharmacy |
| Adult Medicine | RB Physiology |
| Adult Medicine | RC Dental Hygiene |
| Adult Medicine | RE Physiological Measurement |
| Adult Medicine | RF Prosthetics/orthotics |
| Adult Medicine | RF1 Prosthetics |
| Adult Medicine | RF2 Orthotics |
| Adult Medicine | RG Dispensing optometry |
| Adult Medicine | RH Optometry |
| Adult Medicine | RJ Orthoptics |
| Adult Medicine | RK Diagnostic radiography |
| Adult Medicine | RK1 Electroencephalography |
| Adult Medicine | RK2 Electrocardiography |
| Adult Medicine | RK3 Ultrasonics |
| Adult Medicine | RK4 Nuclear medicine |
| Adult Medicine | RL Therapeutic radiography |
| Adult Medicine | RM Medical photography |
| Adult Medicine | RS Dental therapy |
| Adult Medicine | RT Pharmaceutical Medicine |
| Adult Medicine | RU6 Acupuncture |
| Adult Medicine | RU7 Bowen Therapy |
| Adult Medicine | T1 General nursing |
| Adult Medicine | T11 School nursing |
| Adult Medicine | T5 Community nursing (district nursing) |
| Adult Surgical specialties | C1 General Surgery |
| Adult Surgical specialties | C11 General Surgery (excl Vascular) |
| Adult Surgical specialties | C12 Vascular Surgery |
|  | |
| Reports\\SMA hospital specialty caetgory Final | Page 3 of 6 |
| 04/03/2020 17:44 | |
|  | |
|  | |
| **Name** | **Coded Text** |
| Adult Surgical specialties | C13 Oral and Maxillofacial Surgery |
| Adult Surgical specialties | C14 Major Trauma |
| Adult Surgical specialties | C2 Accident & Emergency |
| Adult Surgical specialties | C3 Anaesthetics |
| Adult Surgical specialties | C4 Cardiothoracic Surgery |
| Adult Surgical specialties | C41 Cardiac Surgery |
| Adult Surgical specialties | C42 Thoracic Surgery |
| Adult Surgical specialties | C5 Ear, Nose & Throat (ENT) |
| Adult Surgical specialties | C6 Neurosurgery |
| Adult Surgical specialties | C7 Ophthalmology |
| Adult Surgical specialties | C8 Trauma and Orthopaedic Surgery |
| Adult Surgical specialties | C9 Plastic Surgery |
| Adult Surgical specialties | CB Urology |
| Adult Surgical specialties | D3 Oral Surgery |
| Adult Surgical specialties | D6 Restorative Dentistry |
| Adult Surgical specialties | D61 Restorative Dentistry - Endodontics |
| Adult Surgical specialties | D62 Restorative Dentistry - Periodontics |
| Adult Surgical specialties | D63 Restorative Dentistry - Prosthodontics |
| Adult Surgical specialties | DC Surgical Dentistry |
| Adult Surgical specialties | DD Fixed & Removable Prosthodontics |
| Adult Surgical specialties | R11 Surgical Podiatry |
| Adult Surgical specialties | R7 Ambulancemen/women - Accident & Emergency |
| Adult Surgical specialties | RD Dental Surgery Assistance |
| Adult Surgical specialties | RP Paramedics |
| Hospital specialty categories for SMA |  |
| Maternity and reproductive health services | E11 GP Obstetrics |
| Maternity and reproductive health services | E12 GP Other than Obstetrics |
| Maternity and reproductive health services | F1 Obstetrics & Gynaecology |
| Maternity and reproductive health services | F1A Well Woman Service |
| Maternity and reproductive health services | F1B Family Planning Service |
| Maternity and reproductive health services | F2 Gynaecology |
|  | |
| Reports\\SMA hospital specialty caetgory Final | Page 4 of 6 |
| 04/03/2020 17:44 | |
|  | |
|  | |
| **Name** | **Coded Text** |
| Maternity and reproductive health services | F3 Obstetrics |
| Maternity and reproductive health services | F31 Obstetrics Ante-Natal |
| Maternity and reproductive health services | F32 Obstetrics Post-Natal |
| Maternity and reproductive health services | F4 Community Sexual & Reproductive Health |
| Maternity and reproductive health services | T2 Midwifery |
| Maternity and reproductive health services | T21 Community Midwifery |
| Mental health services | G1 General Psychiatry (Mental Illness) |
| Mental health services | G1A Community Psychiatry |
| Mental health services | G2 Child & Adolescent Psychiatry |
| Mental health services | G21 Child Psychiatry |
| Mental health services | G22 Adolescent Psychiatry |
| Mental health services | G3 Forensic Psychiatry |
| Mental health services | G4 Psychiatry of Old Age |
| Mental health services | G5 Learning Disability |
| Mental health services | G6 Psychotherapy |
| Mental health services | G61 Behavioural Psychotherapy |
| Mental health services | G62 Child & Adolescent Psychotherapy |
| Mental health services | G63 Adult Psychotherapy |
| Mental health services | R2 Clinical psychology |
| Mental health services | RU Arts Therapies |
| Mental health services | RU1 Art Therapy |
| Mental health services | RU2 Drama Therapy |
| Mental health services | RU3 Music Therapy |
| Mental health services | RU4 Dance Therapy |
| Mental health services | RU5 Mistletoe Therapy |
| Mental health services | RU8 Counselling |
| Mental health services | T3 Mental health nursing |
| Mental health services | T31 Community psychiatric nursing |
| Mental health services | T4 Learning disability nursing |
| Mental health services | T41 Community learning disability nursing |
| Paediatrics | A21 Paediatric Cardiology |
|  | |
| Reports\\SMA hospital specialty caetgory Final | Page 5 of 6 |
| 04/03/2020 17:44 | |
|  | |
|  | |
| **Name** | **Coded Text** |
| Paediatrics | AF Paediatrics |
| Paediatrics | AFA Community Child Health |
| Paediatrics | C91 Cleft Lip and Palate Surgery |
| Paediatrics | CA Paediatric Surgery |
| Paediatrics | D8 Paediatric Dentistry |
| Paediatrics | J9 Paediatric and Perinatal Pathology |
| Paediatrics | T6 Health visiting |
| Paediatrics | T7 Sick children's nursing |
| Paediatrics | T8 Nursery nursing |
|  | |
|  | |
|  | |
|  | |
|  | |
|  | |
|  | |
|  | |
| Reports\\SMA hospital specialty caetgory Final | Page 6 of 6 |
