# Supplementary material for: ‘Missingness’ in health care: Associations between hospital utilization and missed appointments in general practice. A retrospective cohort study
Source: PLoS One. 2021 Jun 24;16(6):e0253163. doi: 10.1371/journal.pone.0253163 (PMC8224850; doi:10.1371/journal.pone.0253163)
Supplement: Attachment — Submitted filename: Missingnesshosputilisationrebuttalletter.FC.24.11.2020.docx [file pone.0253163.s002.docx]

** November 2020

Dear PLOS One editorial team.

Thank you for considering our manuscript for publication following its transfer from PLOS Medicine. Please see our point by point consideration of the reviewer’s feedback below.

We found the reviews helpful; we have been able to clarify some key points and improve the narrative of the paper; especially from an international readership perspective. We have clarified and made clearer some analysis decisions and have addressed all reviewer comments.

Changes made in the manuscript are highlighted.

**Reviewer #1:**
**In the abstract the neg bin regression model is described as 'binomial regression', this might be better rephrased as this would typically be used to describe something like a log-binomial regression model of a binary outcome as opposed to the count outcomes you have in this study.**

Thank you for pointing this out. We have changed the wording of the abstract (line 33) to be more accurate.

**To clarify, the exposure (number of missed appts) is determined from Sep 2013 to Sep 2016, and there was inpatient and outpatient data for the outcomes from 2013 to 2017? Was all the outpatient data during this period used? Could someone have missed appointments (and then be classified as a high level of the exposure) after they had had a hospital admission? The temporal relationship is mentioned in the study limitations but there does seem to be a plausible reversal of the association between the exposure and outcome here if they are both ascertained from the same time period (e.g. someone has a health event (recorded as admission) that then means they have more difficulty in keeping subsequent GP appointments).**
We can confirm all the outpatient data from this period were used. We now clarify in the limitation section, line 285-290 that we could not measure temporal relationships in this study. Further explanation is given when responding to the subsequent reviewer question about modelling used.

To provide context to our operationalising of this data and analysis; specifically, scheduling of GP appointments is under the control of the patient, so patients can both schedule and cancel GP appointments according to their need and wishes. That patients who miss multiple GP appointments are higher users of hospital care is an important finding in itself, as we say signalling high treatment burden (further explanation of this term described in line 103), and we suggest requiring a systems response to address this. We also make no assumptions about causality and it could be the case that more admissions lead to higher GP missed appointments. That in itself when considering a whole systems approach is important. Wording has been changed in line 330-331 too to emphasise that a focus on temporality should be a component of future research across health service systems.

**Patients with more healthcare utilisation (and presumably need) would also be more likely to miss appointments simply they had more opportunities to miss appointments (i.e. same underlying risk of missing an appointment, but because they have many more appts scheduled they end up with a higher count of missed appts). Could the association with number of missed appointments and outpatient/inpatient service use just be because those with more underlying need simply had more opportunity to miss appointments? Does the association still hold if the exposure is constructed using the rate of missed appts?**

Thank you. This was a key consideration when devising this study. We adjusted for GP appointments scheduled in the negative binomial modelling and had omitted to include this in our description which we have now rectified, see lines 31-35 and lines 166-167. We opted to account for GP appointments scheduled in the modelling rather than the missed appointments categories, because the modelling takes into account GP appointments scheduled for individual patients rather than by groups if we had used categories. This also reduces the risk of false positive results.

GP appointment scheduling is under the control of patients as mentioned above; we had described that in the original manuscript in lines 123-126 but have now reiterated that in line 107-115.

We previously considered and then rejected controlling for number of outpatient appointments. Outpatient hospital referrals are triggered by the patient’s GP and the appointment scheduling is controlled by hospital services (not the patient) and organisation of this varies by hospital and specialty. Patients do not have open access to specialty hospital care in the UK, only to GP care. Different specialties in different hospitals also schedule differentially – for example a non-attendance in many specialities for a first appointment means the patient is discharged from care; some offer opt in appointments which require additional action by the patient; however this is not uniformly applied and may have changed over the time of the study interval; so we judged it would skew the results markedly if we adjusted for appointments scheduled. This is now explained in the discussion of limitations in lines 292-295.

**P8. L137. What software was used for the analysis?**

Apologies that we missed including this in this paper. We used R version 3.4.0, this is now described and referenced in line 171.

**My experience with similar data (hospital admissions) in a population-based study is that there are often many more 0's than are usually predicted by count (Poisson or neg bin) regression models. Was the distribution of the residuals checked? How if so? A useful way to do this is the oddly named 'hanging rootogram' visualisation.**

The appointments are counts with a high number of zeros. We did attempt to apply more sophisticated modelling to these data but were constrained by the computational capacity of our analysis platform (National Safehaven). A response letter (Ellis et al Lancet Public Health 2018) to an earlier paper from this dataset describes this.

These models were chosen because they are suitable for the available data. The error bars in the forest plots are small which suggest a good fit.

The constraints on computational capacity also explain why we were unable to focus on temporal aspects of this data set. We had chosen for clearly justifiable reasons to analyse a population representative large data set and required to do this within the data handling requirements of NHS Scotland.

**Reviewer #2:** 
**The inability to tell a longitudinal story here is a major weakness and shortcoming of this study. I'm not sure how the authors should address, but the study is functionally a cross sectional analysis with a wide time window. In addition, its highly susceptible to reverse causation. For example, patient who were frequent users of the hospital may be missing GP appointments because they were in the hospital or their medical needs would be better met in the hospital as opposed to the GP's office. So, these patients are missing from their GP's visits. I am concerned that we may be making incorrect inferences that missingness is leading to these events but we can't tell that from this analysis.**
Thank you- see responses to reviewer 1’s comments- we hope this addresses your concerns.

**I'm not convinced that the number of long-term conditions is an adequate substitute or indicator of medical complexity. I'm not familiar with the measure. However, in reviewing the original Barnett et al. article, not all long-term conditions are equal in leading to an admission. Two long term conditions such as hypertension and thyroid disorders are not as likely to lead ot an admission as say heart failure or inflammatory bowel disease. Why not use a comorbidity index that will more appropriately control for disease severity rather than simply the number of conditions?**
Thank you for raising this important point and it is helpful to have the opportunity to clarify this in the paper. There is an established literature on the benefits and drawbacks of measuring multi-morbidity using a range of measures. We chose this particular one because it relates to levels of morbidity relevant to both primary care and secondary care. This is a study about both aspects of care and we wanted to consider treatment burden across both these aspects of the health system. We hypothesise the measure we have used to be a reasonable proxy for the overall treatment burden experienced by patients. We do not assume any direct impact on use of care resulting directly from this measure. We have added this context information at line 149-151 to aid the readers understanding of this.

**I'm not clear that the author's approach using absolute counts of missingness alone, as opposed to relative proportions, of missed GP appointments is the best way to categorize missingness. Patients who have more frequent visits are potentially more likely to miss appointments because they are clinically complex, socially complex, or they prefer to frequent medical appointments. They also have a higher chance of missing an appointment because they have more appointments. As a clinician, I think I would be less worried about any patient who say has 5+ GP appointments in a year and they miss one of those compared to a similarly medical complex patient who has 2 GP appointments in a year and misses 1 of those. They are both missing 1 appointment, but one person is missing at most 20% of their appointments and another person is missing 50% of their appointments.**
**The authors might be able to conduct parallel analyses using percent of visits as the independent variable, or the authors should consider at least stratifying their analysis based on individuals with more frequent or less frequent appointments. For the latter, having some understanding of the distribution of number of GP visits per year may drive how many stratification buckets they could leverage.
Similarly, there is a dynamism to appointment scheduling. Individuals who miss a GP appointment may be able to quickly reschedule those appointments. Those individuals I'm less worried about, clinically, because they are at least trying to engage in care and may have their needs ultimately met. Individuals who miss their appointment but don't show up again for months or years are more worrisome. This should at least be a listed limitation.**
As described above, we did adjust for the number of appointments made in our models. This issue is discussed in our earlier BMC Medicine paper in some more depth (McQueenie et al., 2019). The responses to reviewer 1’s comments address this issue and we have made some changes to the original manuscript to make it clear that missed GP appointments are based on a rate, and we have justified why this was not feasible to follow the same approach for missed hospital outpatient appointments.

**Why would high missed GP be associated with higher attendance at specialties? Perhaps what a GP could offer was being substituted by what a specialist could provide. At that point, it would appear that going to GP is an administrative hassle for the patient who needs to go enough times to receive referrals to a specialist or meet some technical requirement. I'm not as familiar with the NHS to know how/why this may play itself out in the data.**
Thank you for highlighting to us that we need to provide a better explanation for an international audience about how GP versus specialty appointments and attendance works. This has been added at line 107-105 as described above. We have also addressed the related issue of treatment burden in the manuscript (line 103-104).

**Finally, I think some of the discussion or introduction should be spent discussing what the authors think "missingness" represents. Is missingness a distraction from the real problem of poor care (e.g., patients are avoiding that provider) or some socioeconomic need of the patient? Similarly, framing the conversation completely in the context of missingness limits the scope of solutions to solving for fewer missed appointments. Instead, I believe that missing a GP appointment is a sentinel event that may serve as a spark for health care systems to investigate what those patients need to improve their care or have better access to care to begin with**
This is a very important point, and we addressed this issue to a large extent in our earlier paper (McQueenie et al., 2019). We have also added in some text in the introduction- line 79-84 that explains better the reason for our focus on missingness which then hopefully helps contextualise the subsequent theorising we set out in the original manuscript about what missingness actually is in line 86-100.

**The authors use a few terms that are confusing or at least need a better definition for an international audience.**
**1.      Is Trusted Third Party a masked name? Or is that actually the name of the vendor/company?**

Thank you for seeking clarification about this. We have added a definition at line 129-133.
**2.      What is SIMD (Scottish Index of Multiple Deprivation)? I googled it, but I think putting a definition in the text would be easier for the reader.**
A definition and reference has been added at line 168-170.

**Reviewer #3:
According to the authors, their Research Question is "Are multiple missed appointments in general practice associated with increased use of hospital services and missingness from hospital care?". There is not any discussion or data in the text about the use of hospital services; thus an essential part of the research question is not fulfilled.**
We were surprised by this comment. Hospital service use was described in the original manuscript version available to the reviewer. In the current version (amended to take account of the feedback as described in this rebuttal letter) described in the abstract lines 37-40, 42-48, results from line 180-226, and in the discussion line 256-257 and 263-266. We have also structured the abstract to make hospital use outcomes clearer (line 37-40)

**It seems that the authors are not aware of the literature about "missing appointments" or "no-shows". A quick search on Scopus database would return hundreds of articles, but the authors have cited none.**
**The authors proposed "This research is the first to investigate patterns of missed appointments at the patient level and to examine patients' service use across health care in relation to these." Several research pieces have discussed the missed appointments at the patient level; this statement is not appropriate. In reality, the main novelty of the article has already been intensely studied in previous works.**
We disagree with this point. Nevertheless we are grateful to the reviewer for highlighting this and we are perhaps guilty of assuming readers will have read our previous work on this topic. The existing literature about missed appointments is heterogenous and tends to either conflate patients with single missed appointments with those who have many, use small clinic samples, or focus on single disease areas. There is vanishingly little literature on multiple missed appointments within a comprehensive health service. We have added in text in the discussion at line 300-302 to describe this issue in more detail and have cited our previous discussion of this in previous papers. We also hope that having added in line 79-84 aids understanding of our theoretical perspective about this.

**Another question is about the experiment design. The authors classified patients in 4 categories: zero missed appointments; low missed appointments: <1 per year average; medium missed appointments: 1-2 per year average; high missed appointments: >2 per year average. Using these categories, they conclude that "Patients who miss high numbers of GP appointments are higher users of outpatient and inpatient hospital care". As the categories represent the number of missing appointments, it is natural that patients with the largest number of appointments will be more likely to be classified in the "high missed appointments" category. For instance, a patient with just one appointment will never have this classification. Therefore this categorization can mislead the results. In the literature, this question is treated as a percentage of appointments, not in a discrete categorization (remark that in this case, negative binomial modelling would not apply).**

Please see our responses to reviewers 1 and 2 above. We used these categories to enable us to develop the models described in the paper.

[McQueenie, R.](http://eprints.gla.ac.uk/view/author/14838.html), [Ellis, D. A.](http://eprints.gla.ac.uk/view/author/4461.html), [McConnachie, A.](http://eprints.gla.ac.uk/view/author/2254.html) , [Wilson, P.](http://eprints.gla.ac.uk/view/author/10203.html) and [Williamson, A. E.](http://eprints.gla.ac.uk/view/author/1797.html) (2019) [Morbidity, mortality and missed appointments in healthcare: a national retrospective data linkage study.](http://eprints.gla.ac.uk/177481/) [BMC Medicine](http://eprints.gla.ac.uk/view/journal_volume/BMC_Medicine.html), 17, 2. (doi: [10.1186/s12916-018-1234-0](http://dx.doi.org/10.1186/s12916-018-1234-0))

[Ellis, D. A.](http://eprints.gla.ac.uk/view/author/4461.html), [McQueenie, R.](http://eprints.gla.ac.uk/view/author/14838.html), [McConnachie, A.](http://eprints.gla.ac.uk/view/author/2254.html) , [Wilson, P.](http://eprints.gla.ac.uk/view/author/10203.html) and [Williamson, A.](http://eprints.gla.ac.uk/view/author/1797.html) (2018) [Non-attending patients in general practice.](http://eprints.gla.ac.uk/159292/) [*Lancet Public Health*](http://eprints.gla.ac.uk/view/journal_volume/Lancet_Public_Health.html), 3(3), e113. (doi: [10.1016/S2468-2667(18)30020-3](http://dx.doi.org/10.1016/S2468-2667(18)30020-3))
