# Supplementary material for: ‘Missingness’ in health care: Associations between hospital utilization and missed appointments in general practice. A retrospective cohort study
Source: PLoS One. 2021 Jun 24;16(6):e0253163. doi: 10.1371/journal.pone.0253163 (PMC8224850; doi:10.1371/journal.pone.0253163)
Supplement: Attachment — Submitted filename: PLOSone.Response to reviewers.FC.10.02.2021.docx [file pone.0253163.s003.docx]

10/02/2021

Dear Dr Orueta,

**PONE-D-20-37099. ‘Missingness’ in health care: associations between hospital utilization and missed appointments in general practice. A retrospective cohort study.**

Thank you for your positive response to our article. We are grateful for the helpful feedback offered by yourself and the reviewers; and have addressed the comments in turn below.

We note that you have indicated that data from this study are available upon request. PLOS only allows data to be available upon request if there are legal or ethical restrictions on sharing data publicly. For information on unacceptable data access restrictions, please see <http://journals.plos.org/plosone/s/data-availability#loc-unacceptable-data-access-restrictions>.

In your revised cover letter, please address the following prompts:

a) If there are ethical or legal restrictions on sharing a de-identified data set, please explain them in detail (e.g., data contain potentially identifying or sensitive patient information) and who has imposed them (e.g., an ethics committee). Please also provide contact information for a data access committee, ethics committee, or other institutional body to which data requests may be sent.

We will update your Data Availability statement on your behalf to reflect the information you provide.

Here is the statement about data access contained in the manuscript. Do let us know if this is sufficient information.

Data sharing

These data were available from NHS Scotland. Permission for access was granted to the study team only, from the participating GP practices and the Public Benefit and Privacy Panel NHS Scotland. Requests to access data can be made to <http://www.escro.co.uk/> for general practice data and to <https://www.isdscotland.org/Products-and-Services/eDRIS/> to host the analysis of general practice data and for permissions and access to secondary care data. Analysis code is available from the authors upon reasonable request.

Thank you for stating the following in your Competing Interests section: "NO"

Please complete your Competing Interests on the online submission form to state any Competing Interests. If you have no competing interests, please state "The authors have declared that no competing interests exist", as detailed online in our guide for authors at [http://journals.plos.org/plosone/s/submit-now](about:blank)

This information should be included in your cover letter; we will change the online submission form on your behalf.

The authors have declared that no competing interests exist.

Your ethics statement should only appear in the Methods section of your manuscript. If your ethics statement is written in any section besides the Methods, please move it to the Methods section and delete it from any other section. Please ensure that your ethics statement is included in your manuscript, as the ethics statement entered into the online submission form will not be published alongside your manuscript.

Thank you for drawing this to our attention-this has been amended (now at line 137).

Reviewer comments and questions

However, hypothesing missed appointments as a deviating consultation behavior, one would be more interested whether there is an association between missing appointments and some specific LTC like panic disorder, (health) anxiety and other social and psychiatric problems (e.g. homelessness? Schizophrenia?) (Page 12) Your GP database probably contains this information. Why didn’t you included this in your analysis and is it possible to add such an analysis? (R1)

Thank you and we agree there are likely to be underlying LTC drivers and social factors for appointment attendance behaviours. We investigated LTCs and social factors in some depth in our preceding papers using the GP dataset, although of course we can draw no conclusions about causality.

<https://bmcmedicine.biomedcentral.com/articles/10.1186/s12916-018-1234-0> morbidity and mortality outcomes

https://bjgpopen.org/content/4/1/bjgpopen20X101011

ACEs and social marginalisation

Unfortunately although we requested diagnosis data for the linked hospital data, permission to access this was refused by the permissions committee that governs data access in the Scottish NHS. Our study was considered ‘pathfinder’ which means novel and high risk so permissions were restricted to attendance and specialty codes only. Hence we were unable to follow on investigating LTCs and other factors in this linked data.

Does e.g. the Charlson comorbidity index (CCI) not better reflect the burden of diseases than just counting LTC’s? (R1)

The CCI predicts 1-year risk of mortality among general medical patients: it has not been shown to perform well in terms of risk prediction in the general population. We consider that the method used by Barnett et al, on which our multimorbidity score was based, gives a better assessment of the burden of disease in community-based patient groups.

Understanding missing appointments differs according to age category: Missing appointments of the youth can e.g. be explained by self-limiting morbidity, or e.g. depression /addiction which may induce passive behaviour, non-attendance of the frail old can be explained by (aggravation of) existing morbidity and memory problems. Is it possible to perform a sub group analysis of young and old patients? (R1)

We agree that there are likely to be different mechanisms underlying missed appointment behaviour across the lifespan, and this should be the subject of future work. We also have examined this in more detail in the GP data in our previous published paper about patient (and practice) demographics. For the present study we wanted to examine the broad patterns of association between missing primary care appointments and secondary care service usage.

The authors use 4 categories of missed appointments. Why didn’t they use a continuous variable? (R1)

These categories were chosen in order to remain consistent with previous work and allow for straightforward comparisons. We clarify this at line 145.

Reviewer 1 minor points
Does the NHS use SMS to alert patients for a scheduled appointment?

This varies between practices and acute care settings and we were not able to obtain data on this issue. We hope we have explained how heterogenous the appointment support landscape is in line 107.
Abstract: - I miss a (short) description of the ‘why’ (we don’t know whether…. therefore we...)

Thank you- now included in line 23.

page 14: Please describe what you mean by ‘with refinement of codes included in addiction /mental health categories’. Why, what, wherefore?

We examined and refined existing codes for important conditions to confirm the accuracy of mental health and addiction codes. Eg tobacco use related codes were removed. This has been described in line 154
Line 187. Why using SD here instead of CI?

It is usual to report standard deviation (SD) when reporting means. CI’s were used when reporting the RR modelling results.

Line 193-195. Normally one doesn’t draw a conclusion in the result section.
Thank you for drawing our attention to that. The hypothesis statement has been deleted from the results section and is retained in the discussion section only.
Result section (e.g. line 210-2010). I prefer you use one, neutral method of reporting in the result section. I prefer the one you use in line 217-220 without mentioning two-fold, 50% more etc. You better mention your interpretation in the conclusion section.
This paper is for a wide audience so we prefer to also describe the statistically important results in non-statistical terms to enable understanding. We hope this is acceptable.

Line 229. Primary care appointments ore GP appointments?
We agree this may be confusing, so have now amended this to say GP appointments. We have also added in a line at 145 to explain more clearly what this means.

Line 276. I think it would be very interesting to let us know which psychiatric LTC ‘high missers’ have and which LTC ‘no missers’.
Please see comment above about LTC availability of hospital data. We agree!

Line 290. Perhaps it is good to suggest a future prospective cohort research?
That is possible, although we think the evidence (in the UK at the very least) is sufficiently strong to suggest testing interventions which would have an element of prospective data collection for identified patients.
Line 306-308. If I understand your results well, you controlled for age, sex, SIMD, and number of long-term conditions. In that respect I think it isn’t strange you don’t find an association.

We are not sure we understand the question. However the analysis isn’t saying that age, sex, SIMD etc don’t have any association, but that when they are controlled for our conclusions hold.

Line 311-313. I find it remarkable that both patients who (persistently) attend the GP frequently and patients with higher patterns of missed GP appointments shown higher rates of outpatient clinic attendances and hospital admissions. So, deviating consultation patterns of the GP seem to predict use of secondary care.
We agree- two sides of the same coin, there are psychological constructs that may explain this but beyond the scope of this study.
Line 331. Prospective cohort research? In ‘unanswered questions…’ you don’t mention research of the possible financial consequences of missing appointments.

Thank you- we will be factoring economic analysis into future interventions testing research, now amended to mention that, line 345.
Tables.
Please explain all abbreviations (CI, ED etc) in a legend.

Thank you- now updated for Table1-5.

Please consider to mention the P in a legend and not in the table.

We prefer for readability to keep the P values in the table.

I think you better mention ‘Models show relative risk ratio (RRR) and control for age, sex, SIMD, and number of long-term conditions’ in a legend.

We agree on reflection that the captions are unwieldy. We have adjusted the formatting and hope that is now acceptable.
No abbreviations in the title of tables.

This is now amended.

The authors used relative risk ratio in text and relative risk in tables to describe the association between missed appoints in GP and hospital utilization. It will be helpful for the authors to use one measure throughout the manuscript. Since the authors used negative binomial model to analyze hospital utilization, I think it is better to use incidence rate ratio (IRR). (R2)

Thank you for drawing our attention to this. We now consistently use ‘rate ratio (RR)’ throughout the paper to report this.

I disagree with the inclusion of the number of GP appointments scheduled in the model as a covariate. This variable was very likely to be correlated with the main independent variable: missed GP appointments. Instead, the authors can use this variable in defining the main independent variable as a rate (number of missed GP appointments divided by the number of scheduled GP appointments). Zero missed GP appointments means very differently for different numbers of scheduled GP appointments. (R2)

We agree that the number of appointments scheduled is important however, we do not include the number of appointments scheduled as a traditional covariate (line 31, line). Instead, this is included as an offset term. This controls for the issue you rightly highlight, which we have used in previous work. However, this was incorrectly described later in the manuscript and have corrected this on line 173.

As the authors pointed out, the temporal association between missed GP appointments and hospital utilization was not assessed. The outcomes and the main independent variables were measured in the same time period. Thus, the current design could not address the question if missed GP appointments caused higher hospital utilization. A different design may help, for example, one can measure missed GP appointments in the first two years and then examine their association with hospital utilization in the third year. Is this or similar approach feasible? If not, please justify why not. (R2)
The reviewer raises important questions, we agree. This was the first study of this nature that attempted to follow the patient’s journey across health care and was not designed to assess causality, but to explore the epidemiology of patient characteristics and service use. This area is the subject of further research by our team when service usage with temporal relationships will be explored further when seeking to evaluate interventions to reduce missed appointments. In the complex setting of health service use whose rate of uptake is dependent on diagnosed health condition(s), and across multiple settings, the assumptions about temporality and hence causality are challenging.

I am confused with highlighted texts. Are they authors’ notes or are they what they wanted to emphasize? For example, in line 177, “..advised they agreed…”; in line 208, “additional file 1”. (R2)
This reflects changes made when the paper was initially under review at PLOS Medicine. The manuscript was transferred to PLOS ONE directly. These have now been removed.

The authors found a strong association between missed GP appointments and attendance of mental health services and missed appointments in mental health services. I am wondering if mental health issues may have caused missed GP appointments. The authors may consider adding prior mental health diagnosis as a covariate in their models. (R2)

We hope that answers to the question about mental health diagnoses and general limitations regarding causality answer this question.

In lines 292-295, it is confusing. The number of scheduled GP appointments was adjusted in models (line 167), not used in defining the rate of missed GP appointments. But here (line 295), the authors said that the number of scheduled GP appointments was used in defining the rate of missed GP appointments. (R2)
Please see comments above. We have now clarified that the number of GP appointments made was an offset term rather than a covariate.

Some minor problems. In line 145, a period was missing; in line 213, change “four-and-a-half” to “4.5” (and other places alike); in line 234, HMA was defined previously, it was not necessary to define it again; between line 292 and 293, “;” was not properly used for separating nouns. (R2)

Thank you for these helpful comments and have made the changes accordingly. HMA we prefer to define both in the abstract and the discussion to aid the reader’s understanding of terms.

We have uploaded marked and unmarked versions of our manuscript: we consider it has been improved by the reviewers’ input. Thank you again for agreeing an extension to our re-submission time which was compassionate, but in the end did not require.

We look forward to hearing from you in due course.

Yours sincerely

Andrea Williamson,

on behalf of the authors.
