# Supplementary material for: ‘Missingness’ in health care: Associations between hospital utilization and missed appointments in general practice. A retrospective cohort study
Source: PLoS One. 2021 Jun 24;16(6):e0253163. doi: 10.1371/journal.pone.0253163 (PMC8224850; doi:10.1371/journal.pone.0253163)
Supplement: Attachment — Submitted filename: PLOSone.Response to reviewers.2.FC.17.05.2021.docx [file pone.0253163.s004.docx]

17/05/2021

Dear Dr Orueta,

**PONE-D-20-37099. ‘Missingness’ in health care: associations between hospital utilization and missed appointments in general practice. A retrospective cohort study.**

Thank you for your further helpful feedback. We have addressed the comments in turn below. The line number refers to the ‘tracked changes’ version of the manuscript.

1. I don’t fully understand the results. The patients in the group with high missed GP

appointments (HMA) have higher use of outpatient services (RR=1.46). However, when

observing the RR of the 5 categories of outpatient clinics (table-3) all of them are more

elevated than 1.46. Something similar is found in the groups of medium and low missed GP

appointments. This fact is even more patent in the “did not attend” outpatient appointments.

The RRs of any category of the outpatient clinic are higher than the overall RR.

Thank you for bringing this to our attention. We checked the results and the wrong confounding variable was used in the regression model for the SMR00 attendance results. All the rest have been double- checked and are accurate.

The updated SMR00 attendance results are at line 41, and line 211. Table 2 and figure 1 have been updated for outpatient attended data.

Regarding the missed attendance SMR00 data- Table 4 does not have risk ratios that are too high. The reason that these are high is that they are a subset of the appointments for people who did not attend. The 1.46 is just for all appointments, which includes those who did and did not attend. It makes sense therefore that those who did not attend can be above this ratio, as its dragged down to an average of 1.46 when including those who did attend.

2. It is very unusual to mention some limitations of the study in the introduction. Lines 84-91

recognize that observational studies don’t allow to establish causality; lines 93 and following

mention several factors that influence the “missingness” of appointments but some of them

were not included in the analyses; and lines 105-13 describe differences in hospital specialties

organization that can affect the results. Such information is very relevant, but it would be

more appropriate to include it in the discussion.

As it describes the context for why we analyse hospital usage as well as hospital missingness to we think it is important to include the information about preventive care and treatment burden in our introduction. However we accept that the further hypothesising about other factors fits better in the discussion section and have moved this to line 342-350. We have also moved the section about hospital specialities organisation and integrated it into the discussion to line 301-313.

3. In lines 193-9 there is a description of results also presented in table-1. However, such

description is not very accurate. HMA patients received 2.5 times more outpatient

appointments and over 5 times more admissions than those who missed no GP

appointments. This effect is even more pronounced in maternity and mental health

admissions (19.5 and 8 times, respectively).

Thank you for drawing this to our attention and now amended for accuracy in lines 193-199.

4. Lines 211-4. The system to classify specialties into 5 categories should be included in the

methods section, instead of results.

This has been moved to the methods line 166.

Reviewer #1

However, they were not able to solve some issues due to (legal and database) limitations. I think it would be wise to mention this in ‘limitations’.

1.- You state that it is not possible to investigate the association between specific LTC’s (especially

psychological ones like anxiety and panic) and missingness. I think you have to mention this in

‘limitations’. Also, why this wasn’t possible.

2.- You agree that missingness probably is very different for the (very) old and the young with

(perhaps) very different drivers, but it was not possible to perform a subgroup analysis. Please

mention this in the discussion section (limitations).

We examined this in some detail in our previous papers about GP missed appointments- 1 LTCs^[1]^ and 2 demographics^[2]^. Because we were then not given permission to obtain additional hospital level diagnosis data we judged it could be viewed as ‘data mining’ to repeat these analyses with our hospital utilisation data.

3. In the discussion section I miss some words about possible (clinical) explanations of the

associations you found. Personally, I find it remarkable that not only patients who miss GP consultations, but also patients who frequently attend the GP, use more specialist care. As a GP, I think there might be common underlying issues like (health) anxiety, panic and personality. Your

article gains persuasiveness if you explore this briefly in the discussion section. (‘Meaning of the

study…’)

We have emphasised the importance of our previous findings regarding mental health diagnoses in the GP record at line334-335; and trust this also addresses 1 above too.

4. Only knowledge about these possible causal clinical and social factors, can lead to useful

interventions. Please mention which interventions you have in mind. (page 16 line 338)

This section has been expanded at line 362-364.

5. Minor point: In table 2,3, and 4 you state ‘SIMD’. However, in table 5 you state the full description.

Thanks for picking this up- now amended so that this occurs at table 2.

Reviewer #2: Nil to address.

We look forward to hearing from you in due course.

Yours sincerely

Andrea Williamson,

on behalf of the authors.

1. McQueenie R, Ellis DA, McConnachie A, Wilson P, Williamson AE. Morbidity, mortality and missed appointments in healthcare: a national retrospective data linkage study. BMC Medicine. 2019;17(1):2.
2. Ellis DA, McQueenie R, McConnachie A, Wilson P, Williamson AE. Demographic and practice factors predicting repeated non-attendance in primary care: a national retrospective cohort analysis. Lancet Public Health. 2017;2.
